# Healthcare Professionals’ Perceptions about the Implementation of Shared Decision-Making in Primary Care: A Qualitative Study from a Virtual Community of Practice

**DOI:** 10.5334/ijic.6554

**Published:** 2024-04-16

**Authors:** Alezandra Torres-Castaño, Lilisbeth Perestelo-Pérez, Débora Koatz, Vanesa Ramos-García, Ana Isabel González-González, Ana Toledo-Chávarri, Carlos Jesús Bermejo-Caja, Himar Gonzalez-Pacheco, Analia Abt-Sack, Valeria Pacheco-Huergo, Carola Orrego

**Affiliations:** 1 Canary Islands Health Research Institute Foundation, Tenerife, Spain; 2 Evaluation Unit of the Canary Island Health Service (SESCS), Tenerife, Spain; 3 Network for Research on Chronicity, Primary Care, and Health Promotion (RICAPPS), Tenerife, Spain; 4 The Spanish Network of Agencies for Health Technology Assessment and Services of the National Health System (RedETS), Tenerife, Spain; 5 Avedis Donabedian Research Institute (FAD), Spain; 6 Universidad Autónoma de Barcelona, Spain; 7 Innovation and International Projects Unit. Vice-Directorate for Health Research and Documentation. Directorate General for Research, Education and Innovation. Madrid Health Ministry, Spain; 8 Gerencia Asistencial de Atención Primaria, Servicio Madrileño de Salud, Madrid, Spain; 9 Departamento de Enfermería, Universidad Autónoma de Madrid, Madrid, Spain; 10 Centro de Atención Primaria Turó-Vilapicina, Institut Català de la Salut, Barcelona, Spain

**Keywords:** primary care, healthcare professionals, perceptions, empowerment, shared decision-making, virtual community of practice, Atención primaria, Profesionales sanitarios, Percepciones, Empoderamiento, Toma de decisiones compartida, Comunidad virtual de práctica

## Abstract

**Background:**

The incorporation of shared decision making (SDM) is a central part of empowerment processes, as it facilitates greater activation on the part of patients, increasing the likelihood of them gaining control over their healthcare and developing skills to solve their health problems. Despite these benefits, there are still difficulties in the implementation of SDM among healthcare professionals due to internal and external factors related to the context and health systems.

**Aim:**

To explore primary care professionals (PCPs)’ perceptions of the SDM model, based on their preconceptions and experience in clinical practice.

**Methods:**

A framework analysis was conducted on qualitative data derived from a virtual community practice forum, within a cluster-randomized clinical trial developed in the e-MPODERA project.

**Results:**

The most important points in the opinions of the PCPs were: exploring the patients’ values, preferences and expectations, providing them with and checking their understanding of up-to-date and evidence-based health information. The analysis revealed three themes: determinants of the implementation process of SDM, lack of consistency and dilemmas and benefits of PCP active listening, motivation and positive expectations of SDM.

**Discussion:**

In our initial analysis, we examined the connections between the categories of the TDC model and its application in the primary care context. The categories related to the model reflect the theoretical understanding of professionals, while those related to perceptions of its application and use show certain discrepancies. These discrepancies could indicate a lack of understanding of the model and its real-world implications or insufficient commitment on the part of professionals or the organization to ensure its effective implementation.

**Conclusions:**

Specific targeted training that addresses knowledge, attitudes and practice may resolve the aforementioned findings.

## Abstract (es)

**Antecedentes:**

La incorporación de la toma de decisiones compartida (TDC) es una parte central del empoderamiento del paciente, ya que facilita una mayor activación, ganar control sobre la atención que recibe y desarrollar habilidades para resolver sus problemas de salud. A pesar de estos beneficios, todavía existen dificultades para implementar la TDC entre los profesionales sanitarios debido a factores internos de los propios profesionales y externos, relacionados con el contexto y los sistemas sanitarios.

**Objetivo:**

Explorar en el foro de una comunidad virtual de práctica (CVdP) las percepciones de los profesionales de atención primaria (PAP) sobre el modelo de TDC en función de sus ideas preconcebidas y su experiencia en la práctica clínica.

**Métodos:**

Se realizó un análisis de marco desde un abordaje cualitativo de las intervenciones hechas por los PAP en el foro de una CVdP. Esta CVdP se implementó dentro de un ensayo clínico aleatorizado por grupos desarrollado en el proyecto e-MPODERA.

**Resultados:**

Los aspectos más importantes relacionados con la TDC desde la perspectiva de los PAP incluyeron: explorar los valores, preferencias y expectativas de los pacientes, proporcionarles información actualizada y basada en la evidencia, y comprobar su comprensión. En el análisis posterior, tres categorías emergieron como los temas más relevantes: determinantes de la implementación del TDC, falta de consistencia y dilemas, y beneficios de la escucha activa de los PAP, motivación y expectativas positivas de la TDC.

**Discusión:**

En nuestro análisis inicial, examinamos las conexiones entre las categorías del modelo de TDC y su aplicación en el contexto de atención primaria. Las categorías relacionadas con el modelo reflejan la comprensión teórica de los profesionales, mientras que las relativas a las percepciones de su aplicación y uso muestran ciertas discrepancias. Estas discrepancias podrían indicar una falta de comprensión del modelo y de sus implicaciones en el mundo real o un compromiso insuficiente por parte de los profesionales o de la organización para garantizar su aplicación efectiva.

**Conclusión:**

Una formación específica que aborde los conocimientos, las actitudes y la práctica puede resolver los hallazgos mencionados.

## Introduction

Shared decision making (SDM) and integrated care are both important concepts in healthcare and are often interrelated. SDM involves a collaborative process between patients and healthcare providers to make informed decisions about the patient’s care, taking into account the patient’s preferences, values, and needs. Integrated care, on the other hand, involves the coordination of healthcare services across different providers and settings to ensure that the patient receives comprehensive and continuous care [1,2]. The SDM approach can be seen as a key component of integrated care. When healthcare providers work together to coordinate care, they can also work together to ensure that the patient is involved in the decision-making process. This can lead to better outcomes for the patient, as well as increased patient satisfaction and engagement with their care [3]. Patient empowerment is therefore crucial to the SDM process, which occurs when patients accept and take responsibility for their healthcare. One of the main facilitators of patient empowerment is the involvement of healthcare professionals, both in recognising and legitimising the importance of patient self-care and in providing information that enables patients to think critically and make informed decisions about their health [4,5].

In the primary healthcare (PHC) setting, patient empowerment is particularly relevant in the case of chronic diseases, not only because it can improve disease self-management and promote more equitable and collaborative approaches, but also because it can contribute to improved cost-effectiveness in the delivery of healthcare [6,7]. Approaches have been proposed to facilitate the adoption of this model in clinical practice that aim at the practicality of this model. Such is the case of the three-talk model for SDM, which identifies three key moments: the choice talk, the option talk and the decision talk [8]. With this idea of providing support resources to health professionals, decision aids (DA) have also been proposed to support primary care consultations, both for professionals and patients. DA allows them to access balanced, updated and evidence-based information on the main diagnostic and therapeutic options and the risks and benefits associated with each option.

However, despite the benefits of SDM, PCPs have not fully implemented it due to factors related to the context and culture of healthcare [9,10,11]. For instance, PCPs have insufficient training in the communication skills needed to engage their patients in the active process of SDM. Others do not agree with this model due to aspects related to the operationalisation of the clinical situation and patient characteristics [9,12,13].

It has been demonstrated that the participation of PCPs in specific training activities on the SDM model can favour the development of attitudes of approval and improve their perception of self-efficacy in the implementation of this model [14]. Training of PCPs can take place in different formats, with online platforms and virtual communities being one of the formats that have yielded positive results. The results of a case study conducted in 2013 in Spain with healthcare professionals concluded that it is feasible to create a Virtual Community of Practice (VCoP) in PHC that allows for the generation of ideas and innovation in decision making processes [15]. Therefore, the VCoP, defined as a group of people who share a common interest and have the opportunity to deepen their knowledge through continuous interaction in a virtual platform, appears to be a suitable resource to facilitate the learning of SDM among healthcare professionals in the PHC setting [16].

The European Commission has incorporated the concept of patient empowerment and has promoted the development of a series of European projects in the field of eHealth aimed at empowering European citizens to take a more active role in the management of their health. An example of this is the European project EMPATHIE [17], which proposes three interconnected empowerment areas: 1. Health Literacy, 2. Self-management, 3. Shared decision making (SDM).

Combining all the aforementioned elements, the e-MPODERA trial [18,19,20], was developed with the overall objective of improving PCPs attitudes towards the empowerment of patients with chronic diseases (based on the three areas cited above) [17], through their participation in a VCoP for twelve months. This VCoP used discussion forums as the main tool with the aim of sharing experiences, practices and resources to promote collaborative learning among PCPs, share knowledge, raise awareness about patient empowerment and formulate real solutions to specific clinical practice problems.

Based on the data collected in the e-MPODERA project, the present study includes a secondary analysis of the contributions and comments made by healthcare professionals in the VCoP forums. This study aims to explore PCPs perceptions of the SDM model, based on their previous ideas and experience in clinical practice.

## Method

The Standards for Reporting Qualitative Research (SRQR) were followed for the reporting of the results of this study [21].

### Design

A framework analysis was conducted on qualitative data [22]. This approach was used to provide transparency in the process of interpreting the participants’ data, using an analysis matrix composed of previously defined categories and subcategories related to the subject of study [23]. A secondary content analysis [24,25] was conducted of the inputs and comments made by PCPs in VCoP forums within a cluster-randomized clinical trial developed in the e-MPODERA project [20].

These forums provided written resources and discussions for PCPs on patient empowerment. Professionals could interact in the forum asynchronously and freely choose the topics on which they wanted to comment. They had the possibility of replying in some of the forums created by the researchers about the activities proposed in the VCoP. The activities could be content, i.e., relevant information that did not imply an action on the part of the participants, or challenges, which invited them to take an action and to report back in the forums. New contents and challenges related to the empowerment of patients with chronic diseases were proposed on a weekly basis and these activities remained open for twelve months of the VCoP intervention.

#### Framework of the e-MPODERA project

The VCoP activities were designed using a theoretical competency framework based on four learning objectives and twelve core competencies. From these competencies, a series of twenty-six activities were developed for practitioners, of which four activities were related to SDM [18].

The framework used in the e-MPODERA project, based on the four learning objectives and the twelve competencies, can be found in Appendix 1. A screenshot of the VCoP is available in Appendix 4. The activities proposed in the VCoP related to the SDM that can be seen in Table 1.

**Table 1:** Activities of the VCoP related to SDM.


TITLE	OBJECTIVE	PROPOSED ACTIVITY

Understanding shared decisions better and the search for decision aids (DAs).	To explain what DA and their structure are and identify some examples that may be useful.	Identify situations with patients to invite them to share emotions.

Understanding shared decisions better and looking for DAs.	To explore existing DAs.	Find DAs that help them clarify the benefits and risks of different options and people’s preferences.

To treat or not to treat? Making shared decisions.	To reach an informed and satisfactory decision together in a non-real case.	Analysis of a case susceptible to using a DA.

Practising Shared Decision Making.	To analyse the roles of the patient and professional in a clinical interview using the SDM model.	Choose a partner and practice the key elements of SDM.


DA = Decision Aids; SDM = Shared Decision Making; VcoP = Virtual Community of Practice.

### Contextual factors and reflexivity

The research group was composed of experienced researchers (including health services researchers, physicians and psychologists) with experience in the field of SDM. The main interest of the research group was to know the perceptions and experience of PCPs with SDM in order to identify possible barriers to and facilitators of its implementation. The process of reflexivity on the preconceptions that could be conditioning the analysis in the researchers was essential to make these preconceptions explicit and correct possible biases, giving rise to a dialogue that enriched and complemented the jointly conducted analysis. The results are presented anonymously following ethical criteria and the data protection of the participants.

### Sampling strategy

The recruitment of PCPs took place between October 2016 and February 2017, and the randomized controlled trial was conducted during the time that the VCoP was open (March 2017 to May 2018). The participants were recruited from health centres in three regional health authorities in Spain (Canary Islands, Catalonia and Madrid). In each region the research group presented the project in different health centres. PCPs from these centres volunteered to participate and then completed the informed consent. Professionals who had a permanent or substitute position and who did not intend to move during the study period were included.

### Ethical issues

The e-MPODERA Project was approved by the ethics committee of each of the three autonomous communities participating in the project. Before joining the VCoP, the healthcare professionals signed a consent form to participate, after reading an informative letter explaining the project and their participation in detail, as well as explaining aspects related to compliance with confidentiality. Registered on ClinicalTrials.gov 25 April 2016, NCT02757781.

### Data collection

Once the VCoP intervention was completed, the content of the forums was downloaded and the comments of the VCoP participants were reviewed. The information was downloaded in chronological order and was transferred from Drupal 6 to a Word file (Microsoft Office) where a list of comments was created for further analysis and coding.

### Population

A total of 321 professionals participated in the trial, of which 185 belonged to the intervention group and actively participated in the VCoP. Only the comments of 146 PCPs (physicians and nursing staff) specifically related to SDM were considered for the qualitative analysis of the present study. The analysis included a total of 3571 comments made in the forum, which include the moderators’ comments

The characteristics of the health professionals belonging to the e-MPODERA project who participated in the qualitative study can be seen in also Table 2.

**Table 2:** Characteristics of the participants.


CHARACTERISTICS	FREQUENCY

Age (years), mean (SD)	47.03 (8.55)

Sex, n (%)	

Male	31 (21.2%)

Female	115 (78.8%)

Profession, n (%)	

Physicians	80 (54.8%)

Nurses	66 (45.2%)

Resident tutor	

No	112 (76.7%)

Yes	34 (23.3%)

Years of experience, mean (SD)	21.77 (8.08)

Years in primary care, mean (SD)	18.0 (8.28)

Daily caseload, mean (SD)	27.84 (10.68)

Years in the health centre, mean (SD)	8.16 (7.70)


SD = Standard deviation.

### Data analysis

The *framework for the implementation of SDM in primary care* was developed for the analysis of the interventions of health professionals in the VCoP. (A detailed version of this framework can be seen in Appendix 2). Two matrices were used for the development of this framework. The first was the SDM categorization matrix for aged patients with multimorbidity, developed by Vermunt et al. 2019 [26]. This matrix included four main themes: essential elements of SDM, ideal elements, general attributes, and middle ground. The second matrix used was the taxonomy of barriers to and facilitators of the implementation of SDM by Legaré et al. 2008 [9]. This taxonomy includes three main categories: knowledge, attitudes, and behavior, with a series of subcategories identified in each category. The final version of Legaré et al. 2008 [9] considers different factors such as knowledge, attitudes, and behavior that can either act as facilitators or barriers depending on the quality of the professional’s self-efficacy. Table 3 shows an extract from the framework that was adapted for this study.

**Table 3:** The framework used for data analysis.


CATEGORY	DESCRIPTION

Attributes of SDM	This describes the situations that show the general qualities of the SDM model, i.e., Information exchange, deliberation/negotiation, flexibility/individualization, and this involves two people.

Essential elements of SDM	This describes the elements that are considered essential in SDM, i.e., patient values and preferences, definition and explanation of the problem, checking the understanding of the patient, presentation of options, professional knowledge, discussion of the patient’s self-efficacy.

Ideal elements of SDM	Description of the elements that are considered ideal in the SDM model. i.e., mutual agreement, impartial information, presenting evidence, setting goals.

Other characteristics SDM	Description of other important characteristics of the SDM model. i.e., patient education, patient participation companionship, process, mutual respect.

Attitudes	Attitudes of healthcare professionals towards the implementation of SDM. i.e., inapplicability, patient characteristics, clinical situation, lack of general agreement with SDM, lack of expectations on the part of the professional, lack of motivation, self-efficacy.

Behaviour	Behaviours of healthcare professionals towards the implementation of SDM. i.e., factors associated with the organizational culture, time pressure, sharing responsibility with patients.

Knowledge	Health professionals’ knowledge of SDM. i.e., lack of knowledge about SDM, lack of familiarity

Other codes	Paternalistic attitude of the professional

Facilitators	Attitudes and behaviors of the health professional that can facilitate the implementation of SDM. i.e., empathy, motivation.


SDM = Shared Decision Making.

The above framework was the basis of the first version of the codebook. A pilot test was conducted in which three researchers participated by analysing and discussing 200 comments of the PCPs to maintain, dismiss or create new codes.

The coding of the results was conducted using the Framework Analysis (FA) approach to analysing qualitative data. FA aims to classify and organise the data according to key themes in order to develop a hierarchical thematic framework. FA is a case- and theme-based approach, as it allows the combination of data from particular participants and the analysis of data across participants [22]. The codification was supported by NVivo12. Four independent researchers were involved in analysing the comments made in the forums. The final framework was used for the first analysis of the data.

## Results

During the first stage of the analysis, we examined the connections between the categories associated with the SDM model and those linked to its implementation in primary care. The categories linked to the model reflect the professionals’ theoretical understanding of the model, while the categories related to the perceptions of its implementation demonstrate a certain level of inconsistency. These discrepancies could indicate either a lack of comprehension of the model and its practical implications or insufficient commitment on the part of the professionals or the organization towards implementing the model.

Three categories emerged as the most relevant issues: determinants of the implementation process of SDM, lack of consistency and dilemmas, and benefits of PCPs active listening, motivation and positive expectations of SDM.

### Themes

#### Determinants of the implementation process of SDM

This section. refers to a series of beliefs, knowledge and practices of PCPs that may interfere with the implementation of SDM.

Certain beliefs and attitudes have been identified as potential barriers to the adoption of participatory models, such as the SDM approach. One such belief is the notion that responsibility in the clinical relationship primarily rests with healthcare providers. Some PCPs claim that this belief still exists, which leads to paternalistic attitudes. While some PCPs may resort to this approach as a means of expediting decision making during clinical consultations, they acknowledge that it can make patients more dependent in the long term. A listing of the comments and quotations made by the PCPs in the VCoP on the SDM can be found in Appendix 3.

*“The attitudes that professionals sometimes adopt are similar to treating patients like children. I think it is an attitude that we still practise, it due to lack of time, fatigue or the training we have. I also believe that the future of health care involves abandoning these paternalistic attitudes and starting to work in an environment of collaboration with the patient-user, for this we will have to train and change the role that we have always assigned ourselves or have been assigned, at the same time, the methodology of the consultations should also be changed, especially the time that is dedicated to each person.”* (PCP 116)

*“Although we might sometimes disempower, more than that, I believe that we do not insist on empowering them and we have a paternalistic attitude towards patients, especially those who a priori consider that they will not be able to feel empowered and that when you stop a little you see that they are capable and proud to be recognized.”* (PCP 104)

Some PCPs are apprehensive about patient involvement in decision making, as they believe it challenges their authority and autonomy. Other PCPs commented that there are still healthcare professionals who are concerned about a loss of their power and authority when the patient participates in health-related decision making about aspects related to their health. Certain PCPs observed that professionals from the surgical specialties tend to exhibit more paternalistic behaviour, wherein patients are often unaware of the procedures they are about to undergo and do not feel entitled to ask questions.

“*Being a patient in primary care is easier than in specialized care, but we are still far from the ideal fit between patient and healthcare provider. We still do not value the patient enough, their environment, education/training or preparedness to address their chronic pathologies. Many times, I have heard colleagues say I know they will not do it [Improve their health condition], but it is their problem*.” (PCP 48)

“*In my opinion there is a lot of fear of the “loss of power” and this is a big obstacle that we will have to overcome*.” (PCP 20)

PCPs expressed that this perception of power was instilled during their educational years and many providers are more comfortable with it, as it allows them to believe that they hold the truth and the final say in clinical relationships. Some PCPs believe that both providers and patients perpetuate paternalistic relationships although they say assert that this phenomenon is becoming less common.

“*And the work is not only with our patients, we should start with ourselves because all this requires a big change in mentality and way of working, and not all health professionals are willing to leave this “comfort zone” enjoying the privilege of being the one in possession of the information and the one who dictates the rules in the healthcare-patient relationship*.” (PCP 20)

*“In any case, I believe that even today this is a role that is very much incorporated and, although little by little, and especially those who are getting on board, tend or try to change it, it is still the majority case and that it will take time to be able to change, above all the mentality of many “old school” professionals.”* (PCP 37)

PCPs share the idea that SDM requires a more equitable and participatory clinical relationship which should be comprehensive/adequate information exchange and professional dedication to the individual patient. One of the PCPs explains the value of providing comprehensive information and educating the patient as follows:

*“I use the computer to explain the X-ray images to patients or to see lesions on the skin of the process they present. This, while I explain what it is about, reassures them and gives me the feeling that they leave more satisfied. For me it is a positive reinforcement to feel that the patient leaves without uncertainties.”* (PCP 98)

However, comprehensive information and dedication to each patient requires time and some PCPs believe that lack of time hinders this approach. The daily workload they face leaves them with little time to delve deeper into the clinical consultations and encounters and the personal needs and potential of each patient.

*“Yes, sometimes the lack of time means that we are more paternalistic, and this disempowers them, because we solve the most immediate problem, but in the long run what has an impact on improving health is empowerment.”* (PCP 19)

*“But many times, due to lack of time, we anticipate what they want to tell us and we act based on what we believe.”* (PCP 31)

Some professionals also expressed that there is a lack of knowledge and comprehensive understanding of certain steps involved in SDM, which may be a potential barrier to its implementation. They believe that more specific training in the SDM model and more dedicated time with each patient are necessary to overcome their low self-efficacy for implementing the SDM model.

*“It is useless to be full of knowledge if later we are not able to transmit it effectively.”* (PCP 125)

*“It is true that in order to completely address the disempowerment of patients, we need more training and more time to dedicate to each person who attends our practice.”* (PCP 108)

Some PCPs find it challenging to adopt this participatory model during clinical consultations as they lack the necessary skills to motivate their patients to play a more active role. Some PCPs view this as a lack of motivation on their part saying that PCPs need to be motivated themselves to effectively encourage patient participation.

*“I understand that, in order to achieve a greater involvement of patients in their illness or disease, apart from their awareness, it depends a lot on our attitude; I mean our ability to persuade them to make their own decisions to try to improve their health. Therefore, high motivation in the professionals is needed to empower their patients, to successfully transmit this motivation to their patients.”* (PCP 42)

To sum up the main points of the theme, the determinants referred to by the PCPs for implementing SDM regard the professionals’ individual attitudes towards the fundamental values of SDM, the level of professionals’ SDM knowledge, training and self-efficacy, and adequate time to practice SDM in the clinical consultations.

#### Lack of consistency and dilemmas

In this section, we have grouped those categories that point out preconceptions or insights of the professionals that may hinder the implementation of SDM. Notions that do not favour the SDM implementation but that can become more favourable to this model through specific training and increased awareness.

There is a mismatch between the professionals’ perceptions of the SDM theoretical model and their views on its implementation. According to these preconceptions they know all the elements of the SDM model, and some professionals say they have incorporated some elements in their clinical practice. For example, most healthcare professionals define the information exchange between patients and professionals as being of great importance. The shared deliberation process, where both parties present their own perspectives, helps patients understand their disease and treatment possibilities, and helps professionals keep up to date with the available evidence about the disease in question and its treatments.

*“Communication with our patients is very important, understanding each other and making sure that they understand our instructions is essential for the successful treatment of the health condition We have to use easy language … We have to corroborate at all times that the patient understands us and sometimes we have to go through more everyday examples so that the patient understands what we are advising or saying to them. The art of communicating is part of our profession and it is a fundamental pillar to achieve goals with our patients.”* (PCP 59)

Some healthcare professionals say offering person-centred care and exploring patient preferences implies greater knowledge of patients’ individuality, to reorient the clinical relationship and adjust it as much as possible to their reality. Others also mentioned that checking that patients have understood their recommendations is a key aspect. Therefore, they think it is vital to use a checklist to corroborate that patients have understood what the healthcare professional has said to them. It was also observed that patient education was one of the topics that generated the most interest in the VCoP, as it was considered the first step in patient empowerment.

*“Yes, it is very important to make eye contact and make sure that the patient has understood the information we have given them and that they analyse it. We accompany them in that analysis.”*. (PCP 19)

*“It is fundamental to educate the patients to take care of themselves, take care of others and train others. A real chain reaction …”* (PCP 51)

PCPs widely commented on the patients’ use of the Internet, and how it could become a tool for their self-management of the disease, as long as it is supervised by PCPs. Without supervision, the PCPs perceived the use of the Internet as a threat. Other PCPs said that increasing the level of information may lead to the risk of increasing confusion in patients

*“I always tell them” If you don’t know what you are looking for, you don’t know what to find”… there is too much information and they need a guide to know where they can consult. I encourage them to explore, but with caution … because sometimes too much information “misinforms” the patient. We must help them and give them websites to consult.”* (PCP 27)

Most PCPs say that their knowledge of scientific evidence is their main resource for empowering patients. Some PCPs mention that they can help patients’ activation toward self-efficacy when they propose educational interventions and encourage patients to be involved in decision making. These PCPs believe that the patients themselves are the managers of their health and the professionals are mere facilitators of this process. Some PCPs mentioned the benefits of patient co-responsibility for the clinical relationship and for obtaining better clinical results.

*“In my opinion, clearly the patient should be able to assume that the care of their health is a right and an obligation, that it is their responsibility and that we are here to train and help them, offering them all the possible tools that we have at our disposal for this, sometimes with simple interventions and other times more complex ones, but always with their knowledge and approval.”* (PCP 56)

*“This is about an agreement between the two parties, health worker and patient. A shared decision making. It is obvious that if the patient does not want to do something and it is not explained well and they are not persuaded, they will not do it by imposition.”* (PCP 80)

However, even though all their comments, described above, reflect a clear understanding of the shared decision-making model, we found that, at the same time, many PCPs expressed doubts regarding the feasibility of fundamental aspects of the model due to various factors such as the characteristics of the patients and the clinical situation. Concerning the active involvement of the patients, they consider that this involvement or participation is not always possible. Some PCPs said that many patients preferred a clinical relationship, in which the healthcare professional tells them what to do and how to do it. According to this belief, some patients feel more comfortable and it is what most patients have always had, instead of developing their autonomy and being co-participants in decision making.

*“I really like that self-care, self-management and the promotion of the patient’s own responsibility for their health care are promoted. The caveat could be that not all people have the motivation, the necessary knowledge, and that there are generational and cultural differences that hinder the empowerment process.”* (PCP 78)

*“On the other hand, I share the vision of many colleagues, since to change this reality we should involve the patient, because, today, many patients prefer a dependency relationship that frees them from their responsibilities in the face of their disease.”* (PCP 45)

Some PCPs also said that encouraging the involvement of patients, explaining different aspects of the treatments and the disease to patients requires a great effort on their part, since this implies that they should be up to date with the latest scientific evidence and be trained in specific skills, such as active listening. We found, however, that none of the professionals reported using decision aids with their patients to facilitate dialogue with their patients or to present them with up-to-date information on the diagnostic or treatment options about which a decision needs to be made.

Organizational factors surrounding the clinical encounter were also mentioned as important prerequisites, e.g., the need to record patient data in different formats and places (digital databases and on paper) combined with time pressure make it difficult to incorporate more participatory practices.

*“I think that everything helps to disempower our patients, among other things, the increase in Information and Communications Technology (ICT) that has led to a certain dehumanization of the consultation. We pay more attention to registering data and information of the patient in several places and this means that we do not get to hear the complete message of the users and if we put this together with the scarce 10 (minutes) that we have per consultation … everything has an influence in some way.”* (PCP 87)

*“Professional practice, carried out with time management difficulties, can often lead us to be very directive in the intervention, ignoring the capacities to participate that the patient has in the control and treatment of the patient. In addition, it is true that after that dizzying feeling that sometimes directs our consultations, getting on the pulpit is a strong temptation. It is clear that these two attitudes are diametrically removed from the participatory concept that most of us have in caring for our patients. ”* (PCP 41)

Although PCPs may understand and accept the SDM model from a theoretical perspective, when reflecting on its implementation in primary care clinical practice, they encounter conflicting ideas that suggest a lack of understanding regarding the practical implications of shared decision making. Furthermore, they may not fully recognize the potential benefits that the adoption of this model can have in terms of improving health outcomes for their patients and for the overall healthcare system.

#### Benefits of active listening, motivation and positive expectations of SDM

This section includes those attitudes, beliefs and behaviours of PCPs that may support the implementation of SDM in primary care. One which was mentioned was the PCPs capacity for empathy, which they describe as listening and getting to know the patients’ circumstances, being aware of non-verbal communication, putting themselves “in their shoes” and using certain gestures during the consultation. Recording an event in the patient’s life in their medical history and referring to it in the consultation helps to create a “good atmosphere” and a closer clinical relationship. An empathetic attitude, a lot of listening and the use of non-technical language are mentioned as effective ways to facilitate good communication with patients. Furthermore, the more motivation and level of commitment conveyed by the healthcare professional, the more likely the patients are to become involved in their healthcare.

*“I think it is very important to be on the side of the patient and “observe” yourself. I always try to empathize and give a warm smile so that the relationship with the patient is more comfortable. Listening and making them aware that you are listening, this is the most important thing for me.”* (PCP 27)

Some PCPs think that their level of motivation could be improved by training. They believe there is currently a better attitude among healthcare professionals towards patient involvement and that a large majority of PCPs understand the importance of leaving paternalistic models behind and moving towards empowering their patients.

*“It is true that the lack of time often prevents you from dedicating yourself to planning educational groups, but I agree with you that there is a lack of motivation on the part of the professionals, most of the time a high level of motivation leads to a good result.”* (PCP 59)

Some PCPs said that feeling motivated and having positive expectations of patients leads them to be more empathetic and responsive to patients’ expressed information needs. *“I agree that when the patients participate, they make their own decisions about their illness, always or almost always, everything improves.”* (PCP 84)

*“Over the years I have become more aware of the importance of being empathetic. The objective is to establish a trusting, frank and respectful relationship with the patient. The experience with your doctor should be positive from a human and technical point of view. To achieve this, I try to dedicate “all the time” necessary to each patient. It can have some drawbacks: delays in the consultation and more time dedicated to attendance. However, it more than compensates.”* (PCP 80)

*“I totally agree. Informed patients, capable of making decisions and managing their disease help to improve the health system and their own health. We would reduce waiting lists; we would decongest accident and emergency departments.”* (PCP 16)

*“When one is aware of the objectives, and knows and controls the disease, relying on the doctor for decision making, this does not only provide benefits at the health level, but also means the patient is aware and takes part at the level of health spending, and control of resources.”* (PCP 133)

The positive expectations of PCPs regarding the implementation of the SDM model have been emphasized. These expectations are defined as a chain reaction: a better-informed patient can make better decisions and more effectively manage their disease. This can result in improved health outcomes for patients, as well as better functioning of the healthcare system. Specifically, it can reduce waiting lists and alleviate congestion in emergency departments by decreasing the number of medical consultations required. This benefit has been observed in the case of patients with chronic diseases. Some PCPs view empowering their patients as an investment in their relationship with them. By empowering patients, healthcare professionals make them co-responsible for their own health, resulting in positive changes in lifestyle, better adherence to treatments, and improved disease management.

## Discussion

By analysing the perceptions of PCPs towards the implementation of the SDM model in primary care, we identified a number of important themes, such as: determinants of the implementation process of SDM (PCP’s beliefs, knowledge and practices), lack of consistency and dilemmas that could potentially interfere with the implementation process, and benefits of active listening, motivation and PCPs positive expectations of SDM.

From a theoretical perspective of SDM, most professionals believe and state that the SDM approach is already practised in their clinical encounter. They say that effective communication is necessary to establish a close and trusting relationship, which can lead to improved adherence, lifestyle changes, and compliance with the therapeutic plan. Therefore, it is crucial to have conversations in plain language without technical jargon in the clinical setting, and to encourage patient participation, which is in line with general arguments in the SDM literature [5,10,27,28,29].

Although most of the PCPs accept the theoretical aspects of the SDM model, they express limitations when it comes to putting it into practice. They point out that presenting diagnostic and treatment options to patients can be demanding because it requires them to be up to date with the latest scientific evidence. However, few of them are aware of and use DA in clinical practice, indicating that they do not have a detailed understanding of the resources of SDM model they could incorporate. DA can be a support resource in primary care consultations, both for professionals and for patients. DA allows them to access balanced, evidence-based information on the main diagnostic and therapeutic options and the risks and benefits associated with each option [30].

Other determinants of implementation such as PCP’s attitudes and knowledge are related to the lack of consistency between the theoretical perception and the practice of SDM, this is evident when some professionals said that more collaborative and participatory clinical relationships undermined their authority, or when they affirmed that certain patients, due to their limited health literacy or cultural traits, could not be involved more actively in the dialogue. These attitudes suggest a lack of comprehension regarding a crucial aspect of SDM, which involves establishing a shared goal and promoting teamwork between patients and healthcare providers. The three-step SDM model proposes a practical approach to the model that practitioners can implement in clinical practice. [5,8]. However, low levels of implementation persist due to reasons such as the gap between knowing the theoretical model and translating it into actual practice. Healthcare professionals might lack the necessary training or resources to effectively apply the model, thereby hindering its widespread adoption.

Some aspects external to the professionals can reinforce this lack of consistency between what they say about the theoretical model and the practice of the SDM, such as the excessive caseload that leaves little available time to explore patient needs and preferences. These are aspects that have already been documented in other studies on the barriers to the implementation of the SDM [12,31,32,33,34,35,36]. Recent evidence shows that organizational support is a crucial element in the successful implementation of SDM. It is not enough for individual professionals to adopt SDM in isolation; the organization as a whole must prioritize SDM and make the necessary adjustments to support its implementation. The culture and traditions within healthcare systems may not readily embrace a shift towards SDM. Institutional resistance or entrenched hierarchical practices can impede the incorporation of new approaches, including patient involvement in decision making [36,37]. Additionally, incentives and policies within the healthcare system may not sufficiently promote or reward the adoption of shared decision-making. Without appropriate incentivization and policy support, healthcare professionals may not prioritise incorporating shared decision making into their routine practice

Other external aspects are those related to the characteristics of the patients, traits such as low educational level, low health literacy, age, local language barriers, and even those who simply prefer not to engage. PCPs in the study reported that they believed that patients often were unwilling or unable to participate actively in their healthcare decisions. This belief may stem from patients thinking that asking questions or challenging their doctor’s advice is inappropriate. However, these ideas can be altered with the provision of alternative support as DAs or other professional support such as nurses or other health professionals. The SDM model highlights the need for resources and support to help patients participate in their healthcare. Therefore, it is essential to develop interventions that promote patient involvement in decision making and provide them with tools to empower them. Bridging these differences effectively to engage patients in the decision making process is critical for successful implementation [38].

This lack of consistency between the professionals’ perceptions of the SDM model and its implementation has little to do with the results formulated in some studies where regular implementation of the SDM model has been achieved and is practised by dedicated and trained professionals. According to the experience referred to in these studies, it is not so much about making decisions, but rather about offering patients emotional and practical support and helping them to prepare for the next steps in decision making. [39]. In order to do this, it is necessary to keep professionals highly committed to this and to develop relational competencies and receive specific training in the SDM model implementation. Additionally, the integrated care perspective is crucial because healthcare providers should work in a multidisciplinary way and collaborate with one another to ensure that care is coordinated and patient-centred [3,40].

Finally, a key factor for a successful implementation of SDM could be the awareness of professionals and patients in the use of SDM and its benefits. Even when DA are unavailable or unused, fostering awareness of emotional and practical support is associated with better performance in important aspects of SDM, such as informing patients or discussing their preferences [22,40]. However, awareness-raising in SDM does not seem to be an infallible guarantee of genuine integration of empowerment in some population groups or situations [41]. Other key elements are necessary for awareness-raising to help improve the SDM process, such as training for healthcare professionals, communication and relationship building, cultural sensitivity and tailoring, policy and organisational support among others [14,41]. In this respect, motivation and empathy could be awareness-raising drivers, as expressed by the PCPs in this study. Motivation drives them to seek better ways to improve patient care and clinical outcomes. Empathic behaviours enable healthcare professionals to understand the patient’s perspective, needs and preferences, which can lead to a greater awareness of the value of collaborative decision making in clinical practice. These aspects have already been reported as one of the most frequent facilitators of SDM implementation among professionals [9,41]. On the one hand, motivation could arise from the conviction that the SDM model will lead to better health outcomes for patients, as well as better results in the clinical process.

The PCPs also consider the development of their motivational and empathy skills as both a positive aspect and an ongoing challenge. Motivation is directly linked to awareness and understanding. Healthcare professionals need to be aware of the principles, benefits, and processes of SDM to recognize its importance and integrate it into their practice. An awareness campaign highlighting the advantages of SDM could enhance motivation by demonstrating how SDM can improve patient outcomes, enhance patient satisfaction, and foster a more patient-centred approach to care [41].

Regarding empathy, it has been previously reported that those professionals who are not authoritarian or paternalistic, who listen to their patients, who respect their concerns and who create a positive companionship climate with them, make their patients feel more comfortable with open communication and engage more in the clinical relationship [38]. Specifically, motivation implies the dedication of healthcare professionals when providing care to actively engage with patients throughout the healthcare process. This motivation is essential to foster positive interactions, understand patients’ needs and concerns, which is essential in a shared decision-making approach, and improves patients’ overall healthcare experience. It is therefore important to intensify awareness campaigns, both for patients and healthcare staff, on the resources needed to reinforce motivation and empathy during the healthcare process.

In any case, SDM implementation needs to recognise that, in some settings, not being involved in decision making could be culturally acceptable and, in certain circumstances, may be desired by the patients themselves [42]. Based on the results of the present study, we believe that a more detailed contextual analysis would provide more complete information to formulate interventions based on the SDM model that are culturally sensitive and that favour a more flexible and adaptable patient-professional relationship in different care settings [43,44,45,46]. This would significantly improve SDM implementation in primary care settings.

## Conclusions

PCPs said that some aspects related to the shared decision-making process, such as: exploring patient values, preferences and expectations, providing them with up-to-date and evidence-based health information, and validating their understanding were the most relevant aspects related to the SDM approach. However, on closer examination of their views on implementation, we uncovered ideas, beliefs, and preconceptions among these PCPs) that revealed certain contradictions and obstacles within clinical practice. Addressing these through targeted training covering knowledge, attitudes, and practices could effectively enhance PCPs’ awareness, which is in line with the findings observed. Furthermore, effective SDM implementation requires a holistic approach involving all stakeholders, including patients, healthcare professionals, institutions, and health policymakers. Without collaboration and a unified effort to overcome these barriers, the integration of SDM into routine clinical practice will remain low.

## Limitations

Being a secondary analysis of data from a clinical trial, it was not possible to explore in depth the experiences of the PCPs in the implementation of SDM in clinical practice and it was not possible to obtain specific information from them on whether they had received training in SDM or had the knowledge and/or used any DA.

Despite the above, it has been possible to collect relevant information on PCPs’ perceptions of the SDM model and its implementation in primary care.

## Additional Files

The additional files for this article can be found as follows:

10.5334/ijic.6554.s1 Appendix 1. Structure of learning objectives and competences of the e-MPODERA project.

10.5334/ijic.6554.s2 Appendix 2. Framework used to analyse PCPs’ interventions in the VCoP forum.

10.5334/ijic.6554.s3 Appendix 3. Selection of quotes made by primary health care professionals in the VCoP Forum.

10.5334/ijic.6554.s4 Appendix 4. Screenshot of the Screenshot of the e-MPODERA project’s Virtual Community of Practice.
