# Supplementary material for: Healthcare Professionals’ Perceptions about the Implementation of Shared Decision-Making in Primary Care: A Qualitative Study from a Virtual Community of Practice
Source: Int J Integr Care. 2024 Apr 16;24(2):8. doi: 10.5334/ijic.6554 (PMC11025573; doi:10.5334/ijic.6554)
Supplement: Appendix 1. — Structure of learning objectives and competences of the e-MPODERA project. [file ijic-24-2-6554-s1.pdf]

Appendix 1. Structure of learning objectives and competences of the e-MPODERA project

**Learning Objective A:** To improve awareness of patients' and carers' empowerment
A1: To know the impact of patient empowerment and disempowerment
A2: To know interventions and their effectiveness to improve patient empowerment
**Learning Objective B:** To improve familiarity with the concepts of empowerment
B1: To identify the dimensions of patient empowerment and skills related to applying them
B2: To know the taxonomy related to patient empowerment
B3: To know the characteristics and the theoretical and conceptual approach of initiatives that work in patient empowerment
B4: To know new models and paradigms of patient-health professional relationships
**Learning Objective C:** To improve perceived self-efficacy and confidence in one's abilities
C1: To apply strategies and tools to facilitate patients' empowerment in daily practice
C2: To identify barriers to empowerment and establish improvement actions
C3: To develop content and materials on empowerment and/or feedback and to contribute new knowledge to the VCoP
**Learning Objective D:** To improve credibility and expectations about favourable results of empowerment
D1: To learn about Spanish, European and international initiatives of leaders or prominent organisations regarding empowerment
D2: To enhance feedback and dialogue with professionals about the empowerment strategies used
D3. To resolve myths and misconceptions about empowerment

VcoP = Virtual Community of Practice.
