# Supplementary material for: Healthcare Professionals’ Perceptions about the Implementation of Shared Decision-Making in Primary Care: A Qualitative Study from a Virtual Community of Practice
Source: Int J Integr Care. 2024 Apr 16;24(2):8. doi: 10.5334/ijic.6554 (PMC11025573; doi:10.5334/ijic.6554)
Supplement: Appendix 2. — Framework used to analyse PCPs’ interventions in the VCoP forum. [file ijic-24-2-6554-s2.pdf]

Appendix 2. Framework used to analyse PCPs' interventions in the VCoP forum

**Attributes of SDM**<br>*This group of codes describes the situations that show the general qualities of the SDM model*	
Information exchange	Situations where an exchange of information occurs.
Deliberation/Negotiation	Description of situations in which the moment of deliberation/negotiation between the patient and the healthcare professional is observed.
Flexibility/individualization	Description of situations in which it is observed that the SDM model is flexible and can be customized
Involves two people	Description of situations in which SDM involves the patient and the professional
**Essential elements TDC**<br>*This group of codes describes the elements that are considered essential in SDM*	
Patient values and preferences	Situations in which the patients' values and preferences are explored by the professional
Define and explain the problem	Description of situations in which the professional introduces and explains the problem or situation which a decision must be made about
Check understanding in the patient	Situations in which the professional validates the understanding of the information in their patients
Present options	Situations in which the professional presents options
Professional Knowledge	Description of situations in which the knowledge of the professional becomes relevant
Discuss the patient's self-efficacy	Situations are presented in which the professional analyzes, presents or explains the self-efficacy of their patient
Discuss pros and cons	Situations in which the professional discusses the pros and cons of the illness or a procedure with their patients
Organize follow-up	Description of how follow-up is proposed in an SDM model
Make or postpone the decision	Situations in which a decision is made or postponed
**Ideal elements SDM**<br>*Description of the elements that are considered ideal in the SDM model*	
Mutual agreement	Situations in which there is agreement between the professional and the patient
Impartial information	Situations in which the information provided is impartial
Present evidence	Situations in which the professional presents evidence about a disease or diagnostic procedure or treatment
Set goals	Situations where goals/targets are set in SDM
**Other characteristics SDM**<br>*Description of other important characteristics of the SDM model*	
Patient education	Situations in which patients are educated
Patient participation	Situations in which it is observed how professionals promote patient participation
Companionship	Situations in which companionship is established between patients and healthcare professionals
Process	Situations in which the parts of the process followed in SDM are observed

Mutual respect	Situations in which respect between professionals and patients is evident
**Attitudes**	
Inapplicability	Attitudes of professionals who do not believe in the applicability of the SDM model
Patient characteristics	Inapplicability of SDM due to patient characteristics. Such as not knowing how to read or write, aged people or any other characteristic
Clinical situation	Situations in which the clinical situation makes it difficult to implement SDM
Lack of general agreement with SDM (Unspecified)	Professional disagrees with SDM, without specifying why
Too rigid/cookery book	Professionals who perceive SDM as a rigid, artificial or detached from reality model
Challenges the autonomy of the professional	Professionals who do not agree with the SDM model because it challenges their autonomy in the clinical relationship
Disagreement with asking the patient about the desired role	Professionals are dissatisfied or disagree with asking the patient about their interest in SDM and the role they would like to have
Distrust in the patients' abilities	When the professional does not trust the abilities of the patients
Lack of expectations on the part of the professional	Professionals who do not have positive expectations that SDM can work
Lack of self-efficacy	The professional does not believe that SDM can be put into practice
Lack of motivation	Lack of motivation of the professional to use the SDM model
Generates expectations	Perception that the use of SDM will bring about expectations
Health care process	Professional perception that SDM will have an effect on the health care of patients
Patient outcomes	Perception of the professional that SDM will have some results in the patient
**Behavior**	
Factors associated with the organizational culture	All the items that have to do with barriers associated with the environment are explored here
Time pressure	Insufficient time to put the SDM model into practice
Sharing responsibility with patients	Perception of professionals that responsibility is shared with patients
Lack of access to services	Lack of access to services to put SDM into practice.
Lack of resources	Insufficient staff or resources to put SDM into practice
Risk of bad practices when adopting SDM	Description of situations of bad practices that occur due to adopting an SDM model
Patient preferences (for other models)	Professionals consider that patients prefer another model
Factors associated with SDM Source of innovation	Professional behaviors that can be barriers because they do not perceive SDM as a source of innovation
Increases uncertainty	Perception that the use of SDM will increase uncertainty
It is not easy to convey	Perception that it is not possible to share information between patient and professional to reach a mutual understanding in an SDM model

**Knowledge**	
Lack of knowledge about SDM	The professional recognizes that they lack knowledge about the SDM model
Lack of familiarity with SDM	Situations in which the professionals openly acknowledge not being familiar with the model
Forgetfulness	Situations of forgetting to implement SDM
**Other codes**	
Paternalistic attitude of the professional	Situations in which the health professional shows a paternalistic attitude
**Facilitators**	
Professionals' empathy	Description of how professionals' empathy is a facilitator of the implementation of SDM
Professionals' motivation	Situations in which the professionals' motivation is a facilitator to implement SDM
Perception that SDM improves the clinical process	Situations in which it is perceived that implementing SDM entails an improvement of the clinical process
Perception of positive impact on patient outcomes	Situations in which it is perceived that implementing SDM facilitates positive clinical outcomes in patients
