# Supplementary material for: Healthcare Professionals’ Perceptions about the Implementation of Shared Decision-Making in Primary Care: A Qualitative Study from a Virtual Community of Practice
Source: Int J Integr Care. 2024 Apr 16;24(2):8. doi: 10.5334/ijic.6554 (PMC11025573; doi:10.5334/ijic.6554)
Supplement: Appendix 3. — Selection of quotes made by primary health care professionals in the VCoP Forum. [file ijic-24-2-6554-s3.pdf]

Appendix 3. Selection of quotes made by primary health care professionals in the VCoP Forum

Themes	Categories	Subcategories	Number of comments	Comments of professionals on VCoP
Shared Decision Making (SDM)				
**Aspects related to the SDM model**	Attributes of SDM	Information exchange	**76**	**P52:** *"Bearing in mind that the basis of empowerment is knowledge; If the patient does not understand their health/illness, they cannot be empowered, that is, any form of lack of communication or understanding between the professional and the patient already entails disempowerment. In other words, the health professional should become a source of health information and know how to transmit it adequately to allow a good understanding and facilitate joint decision-making. Anything that prevents this flow of information, that disables the patient to understand their health problem, also disempowers them."*<br>**P141:** *"I believe that SDM should be an interactive process where joint decisions are made between the professional and the patient. The professional should provide knowledge (risks/benefits of a preventive/therapeutic attitude) while the patient does it from their values, preferences, concerns and experience."*
		Deliberation/ negociation	**42**	**P73**: *" Aspects of negotiation, accountability and companionship can be introduced during a doctor-patient conversation,"*<br>**P143**: *"Any medical act involves a shared negotiation between the patient and the professional with the most accurate information possible, the decision should be shared, after knowing the benefits, risks, other possible options, etc. "*
		Flexibility/ individualized	**36**	**P17:** *"We have always said that all patients are individual and should be treated as such."*<br>**P17:** *"We do not have to inform everyone in the same way, nor should we set the same goals for everyone."*<br>**P76:** *"An attempt is made to explain sufficiently clearly, simply and at a level that corresponds to each patient, so that they can decide whether or not to do something in some cases."*<br>**P64:** *"Sometimes we disempower patients, many times due to lack of time, it is faster to act in this way when there is no time .... Anyway, at present, we are moving towards a customized way of acting with each person, focusing on the person in an individualized way, providing them with tools to make them feel that they are part of an active form of their disease and for me this is a great achievement and I think we should continue along on this path. "*
		Involves two persons	**40**	**P80:** *This is about an agreement between the two parties, health worker and patient. A shared decision making. It is obvious that if the patient does not want to do something and it is not explained well and they are not persuaded, they will not do it by imposition."*<br>**P51:** *"SDM is an interactive process where decisions are made jointly between the healthcare professional and the patient. The health professional contributes their knowledge, as well as information about the risks and benefits of a diagnostic or therapeutic intervention, while the patient does so about their values, preferences, concerns and experience with the health problem."*<br>**P109:** *"At other times I talk to the patient about 'us'. I tell them about the goal we have to achieve, I explain what we have to achieve to improve their health. An example, stepped increase in analgesia for chronic pain. I explain the side effects. How they can increase the analgesia and if they do not tolerate it, how to lower it. The different treatment possibilities. That it may not work, that we should start with another option, etc. We will see each other again in three weeks. what do you think? Obviously, I cannot do this with every patient who comes to the consultation. This takes time."*

Shared Decision Making (SDM)				
**Specific aspects of SDM**	Essential elements	Patient values and preferences	75	**P17:** *"Prohibiting everything to patients, without taking into account their opinions, customs, situations ... is a clear way to disempower."*<br>**P73:** *"I agree that in the end it is always the patient's tastes and preferences that predominate to guarantee correct compliance with the therapeutic plan and avoid claudication."*<br>**P51:** *"SDM is an interactive process where decisions are made jointly between the healthcare professional and the patient. The health professional contributes their knowledge, as well as information about the risks and benefits of a diagnostic or therapeutic intervention, while the patient does so about their values, preferences, concerns and experience with the health problem."*<br>**P34:** *"On more occasions than we think, the beliefs or expectations of our patients are not satisfied by our scientific actions. I think we should learn to value a little more these aspects that have to do with the sociocultural - ethnic birth context."*
		Define and explain the problem	68	**P31:** *"I don't mind if they ask me, although it takes me a long time to attend to them, I like it when they leave the consultation with clear ideas, I also use drawings many times."*<br>**P31:** *"They sometimes ask questions about things that may seem absurd and unimportant, but you have to listen to them and give them answers to what worries them. They have to feel listened to, and that we pay attention to their doubts, since, if not, they will not ask us again ... "*<br>**P98:** *"I use the computer to explain the X-ray images to patients or to see lesions on the skin of the process they present. This, while I explain what it is about, reassures them and gives me the feeling that they leave more satisfied. For me it is a positive reinforcement to feel that the patient leaves without uncertainties "*<br>**P51:** *"The pilot test has been useful to verify that it is not good to give all the information at once, it has to be sequential. A bit of space is also good to verify that it has been understood and to be able to ask questions and resolve doubts and leave an online channel (email) open for follow-up."*
		Check/clarify understanding	56	**P88:** *"No matter how behind I am in the consultation or how little time I have; I always listen to my patients and before they leave, I try to make sure that they understand what I have said."*<br>**P19:** *"Yes, it is very important to make eye contact and make sure that the patient has understood (they have the information and analyze it). We accompany them in that analysis. "*<br>**P4:** *"I also believe that of all the concepts seen in the game and related to health literacy, the most important is the interpretation of the information by the patient and how we health workers (both doctors and nurses) transmit it."*

		Present options	50	**P30**: *"It is a tool and it is essential. Because we shouldn't decide for patients. You have to put the cards on the table and they choose the one they want to play and how to play it."* **P128:** *"The professional facilitates the patient's access to information based on evidence, not on what they think, it gives them alternatives. The patient chooses between the possibilities presented, asking the professional for an opinion if they deem it appropriate. "* **P49:** *"As we have to modify the treatment, we would talk to them about the different options that exist, with the benefits they may have for them, and decide together which one they think may be a better option for them."*
		Health professional's knowledge.	25	**P125:** *"It is useless to be full of knowledge if later we are not able to transmit it effectively."* **P125:** *"A good number of patients frequently ask questions related to things that they have not understood, especially in relation to technical terms. I think it is absolutely necessary that doubts are resolved by us in a natural way and remove the fear that we sometimes have to ask. "*
		Discuss patient ability/self-efficacy	21	**P60:** *"I think there are all colors, but the important thing is to know who they are, when, as you say, what their disease is and what tools we can offer them, or if it is the time to offer them or not"*
	**Other Characteristics**	Patient education	137	**P51:** *"It is fundamental to educate/literate, as they say in the video to learn to take care of yourself, take care of others and train others. A real chain reaction ... "* **P67:** *"Use the method in which each patient performs best: illustration, storytelling, comparisons, examples."* **P27:** *"I always tell them" who does not know what they are looking for, does not know what they find "... there is too much information and they need a guide to know where they can consult. I encourage them to explore, but with caution ... because sometimes too much information "misinforms" the patient. We must help them and give them websites to consult."* **P7:** *"A good way to start is by searching on the internet, for example. I tell them to search, but to try to enter reliable pages. If I can tell them an address where they can find reliable information. I myself go online in the consultation and I show them what I explain to them with images, their pathology a cervicalgia with rectification of the cervical in their x-ray and I look for a normal one on the Internet, for example. "*
		Patient Participation	57	***P54:*** *"Empowerment is co-responsibility and participation. An empowered patient is a patient with the ability to decide about and control their health. Empowerment implies a less paternalistic model based on participation in both decision-making and self-care."* **P124:** *"We empower them by informing and inviting them to participate by giving their opinions and making decisions about their treatments, studies, etc. so that they feel they have more control of their process, unloading much of the anguish and fears that they generate. "*

Barriers and facilitators for the implementation of SDM				
**Attitudes**	Inapplicability	Patient characteristics	102	**P123:** *"I believe that we should effectively change the paternalistic attitude that professionals have had towards our patients until now, agreeing on the informed decisions that the patient should make, and taking responsibility for their problems, I also have to say that not all patients agree with this since their education/training does not help them to make certain decisions and they ask for your help. "*<br>**P57***: "I work in a center in a disadvantaged area. Older people are functionally illiterate and I have to explain to young people the meaning of, for example, "every other day." Although I have colleagues from areas of a higher socioeconomic level; who tell me they are "those who know". This creates a problem for them with Dr. Google. I do not know. I guess everything has advantages and disadvantages. I miss talking and being understood the first time. When I ask, since when it has happened to them, they sometimes answer me with something other than "eh?". I get very tired of speaking slowly, as if they were tourists and I was giving them directions."*<br>**P106:** *"Not all patients want to participate in empowerment, there are many who go to have the annual check-up and that's it. And if there is any altered factor in the tests, see you next year. "*<br>**P48:** *"Being a patient in primary care is easier than in specialized care, but we are still far from the ideal fit between patient and healthcare provider. We still do not value the patient enough, their environment, education/training or preparedness to address their chronic pathologies. Many times, I have heard colleagues say I know they will not do it, but it is their problem."*<br>**P56:** *"It seems like a complex change to me because we are on a slow walk of an absolutely paternalistic model, in which what the health worker says "is law "and the patient simply obeys and let us not forget that in this environment both health professionals and patients have felt quite comfortable. This has led us to create a great dependence of patients towards the health system and its professionals (hence some do not know how to take a step with respect to their health without asking for an opinion from "their referents") and an important comfort in the professionals is that we sometimes see rejecting or losing as difficult."*<br>**P106:** *"You try to motivate the patients to involve them in decision-making to facilitate an objective, and they tell you "that you are the professional, that you tell them what to do and if they have time, they will do it."*
		Clinical situation	44	**P32:** *"I find it very interesting, and I think that more and more, we share the form of follow-up, and we transfer decision-making to users. Time, an action which is sometimes excessively organized, and at other times convenient, means that other times we don't do it."*
				**P41:** *"Professional practice, carried out with time management difficulties, can often lead us to be very directive in the intervention, ignoring the capacities to participate that the patient has in the control and treatment of the patient. In addition, it is true that after that dizzying feeling that sometimes directs our consultations, getting on the pulpit is a strong temptation. It is clear that these two attitudes are diametrically far removed from the participatory concept that most of us have of caring for our patients. "*
				**P51***: "The position of our consultations, the irruption of computers, wearing a uniform, forgetting the greeting and handshake ..."*

				**P113:** *"I feel that we should empower ourselves first, our mental health is important."*
				**P87:** *"I think that everything helps to disempower our patients, among other things, the increase in ICTs that has led to a certain dehumanization of the consultation. We pay more attention to registering data AND INFORMATION OF THE PATIENT in several places and this means that we DO NOT get to hear the complete message of the users and if we put this together with the scarce 10 (minutes) that we have per consultation ... everything has an influence in some way."*
	Lack of agreement with general aspects of the model	Challenges the autonomy of the professional	21	**P20:** *"Being realistic, SOMETIMES, we empower our patients and most of the time we are paternalistic, imposing, directing and we believe we are in possession of the truth."*<br>**P37:** *"In any case, I believe that even today this is a role that is very much incorporated and, although little by little, and especially those who are getting on board, tend or try to change it, it is still the majority case and that it will take time to be able to change, above all the mentality of many "old school" professionals."*
	Lack of profesional expectations	Lack of self-efficacy	13	**P20:** *"And the work is not only with our patients, we should start with ourselves because all this requires a big change in mentality and way of working, and not all health professionals are willing to leave this "comfort zone" enjoying the privilege of being the one in possession of the information and the one who dictates the rules in the healthcare-patient relationship."*
				**P48:** *"I think we have to find a middle ground, talk more with the patient, learn more about their reality and try to adapt the information and treatment to their capacities, needs, or possibilities. It is true that many have little interest, but many cannot, do not know, are not capable ... "*
		Lack of motivation		**P42:** *"I understand that, in order to achieve a greater involvement of patients in their illness or disease, apart from their awareness, it depends a lot on our attitude; I mean our ability to persuade them to make their own decisions to try to improve their health. Therefore, high motivation in the professionals is needed to empower their patients, to successfully transmit this motivation to their patients. "*
**Behaviors (External barriers)**	Factors associated with the organizational culture	Time pressure	67	**P19:** *"Yes, sometimes the lack of time means that we are more paternalistic, and this disempowers them, because we solve the most immediate problem, but in the long run what has an impact on improving health is empowerment."*
				**P72:** *"I completely agree, we have a lot of things to do inside and outside the consultations. We are overloaded, without enough visiting time, long waiting lists, injustices in schedules, brutal differences between professionals ... "*
				**P51:** *"MORE time is required during and between visits. A lot of collaboration between doctors and nurses in common spaces to talk about cases. "*
				**P31:** *"But many times due to lack of time, we anticipate what they want to tell us and we act based on what we believe."*
				**P135:** *"I agree that many of us lack time to be able to do person-centered practice that aims at shared decision-making, but I also think that paternalistic strategies focused on the disease and the professional, without a doubt are faster, but in the long run their results are frustrating (increasingly dependent and hyper-utilizing patients, medicalized, demanding patients with unrealistic expectations and badly controlled. The problem is knowing how and being able to do it better. "*
				**P141:** *"I think that we do not have enough time in consultation, but it is also true that it is always a good excuse not to change. I think it is not necessary to change everything on day one, but it is necessary to start at some point. I believe that the time we invest in helping them to be more independent will have a medium and long-term impact on our sense of well-being in consultations and on our own satisfaction. "*

		Share responsibility with patient	18	**P116:** *"The attitudes that professionals sometimes adopt are similar to treating patients like children. I think it is an attitude that we still practise, be it due to lack of time, fatigue or the training we have. I also believe that the future of health care involves abandoning these paternalistic attitudes and starting to work in an environment of collaboration with the patient-user, for this we will have to train and change the role that we have always assigned ourselves or have been assigned, at the same time, the methodology of the consultations should also be changed, especially the time that is dedicated to each person."*
				**P35:** *"I fully agree that communication and understanding are necessary for good empowerment, but at the same time the resolution of the health problem must be shared, if you understand our proposal, but do not share it, you will surely abandon it."*
	Factors associated with patients	Patient preference	10	**P2:** *"But it is also true that, on occasion, I have met patients who I have tried to involve in decision-making and self-care of their health and they have told me that I was the doctor!"*
				**P66:** *"In rural areas there are probably factors that facilitate working together with the patient (more time, more direct contact with the patient inside and outside the consultation, better accessibility, etc.) allowing them to take responsibility for their health and problems. It is also true that some patients prefer that the doctor tells them what to do at all times."*
**SDM Knowledge**	Lack of knowledge	Lack of specific knowledge of SDM	38	**P49:** *"For me it is also very difficult to empower these patients, because it gives me the feeling that apart from not having time in consultations, they are the typical patients who never have enough and that when one problem is solved, another problem arises and for me it is easier dealing with the known problem than not empowering again every visit. "*<br>**P108:** *"It is true that in order to completely address the disempowerment of patients, we need more training and more time to dedicate to each person who attends our practice."*
**Other topics**	Paternalistic attitude of the professional		19	**P56:** *"We all see this type of patient every day and we continue to see professionals who continue to be paternalistic (although the truth is, I think less and less), possibly we all fall into this more often than we would like; and it is hard for us to fight against it because we have to talk a lot to the patient and give them a lot of explanations and we all know that we have little time. "*<br>**P56:** *"I personally see a lot of paternalism especially even in surgical specialties. How many times do patients come to our consultation already on the surgical waiting list and they come to ask us what they are going to do because in the hospital consultation they have been afraid and they have not been able to ask anything nor have they had enough explained to them or they have not understood well because they have been in a state of shock. "*
**SDM Facilitators**	Professionals' empathy		48	**P78:** *"Disempowering, I think this refers to the fact that, with our actions, care, treatments, when chosen by us and not by the users, we are promoting the loss of autonomy and fostering a paternalistic relationship, leaving it to the professional to take hold of the reins of the patient's health. "*
				**P84:** *"I don't think we will disempower our patients, but we sometimes take the initiative ourselves, rather than the patient taking it. You don't have to have such a paternalistic attitude."*
				**P72:** *"When there is a good atmosphere and empathy, I think the patient notices it right away, communication is easy and treatment improves, follow-up, treatment, etc."*
				**P125:** *"I try to be as empathetic as possible, not to get carried away by the whirlwind of the consultation, the rush, the patients without appointments and the preconceived ideas towards certain patients."*
				**P98:** *"I distinguish between using patient labels and labeling a patient. I use (post-it) tags to remember details that the patient has entrusted me with, which are not relevant in the medical history but which allow me to be closer to the patient when I see them in a following visit, for example, a trip that they were going to make or the illness of a relative or friend ... etc. I realize that if I ask them about it, the relationship at the time improves. I like doing it that way."*

			**P80:** *“Over the years I have become more aware of the importance of being empathetic. The objective is to establish a trusting, frank and respectful relationship with the patient. The experience with your doctor should be positive from a human and technical point of view. To achieve this, I try to dedicate "all the time" necessary to each patient. It can have some drawbacks: delays in the consultation, more time dedicated to assistance. However, it more than compensates."*<br>**P59:** *“The fundamental pillar to achieve a good dynamic for communication is to be empathetic, to listen a lot to the patient and to use a non-technical language. With these three things, we can facilitate communication with the user and thus facilitate the understanding of their pathologies and their treatments. I always let them talk and afterwards it is very important to ask them questions to see if they have understood what we have explained to them. Furthermore, it doesn't bother me that they come to me with lists and ask me questions about things they don't understand if we want to empower the patient. The first step is for them to be aware of their pathologies and treatments and not to have any doubts. "*<br>**P125:** *"There is no better way to see things than to get under someone else's skin, Empathy!"*
	Professionals’ motivation	40	**P80:** *“Shared decision making. Patient and clinician collaborating together on the patient's problems. We help the patient to find the intellectual, emotional and practical motivation to start on the solution of their problem. "*<br>**P1:** *"Those of us in primary care have the opportunity to be closer to the patients and to help them make decisions, with the patients being active subjects of the decisions and not passive ones."*<br>**P31:** *“Every day I try to empower my patients, and like me other colleagues, more and more, and I think fairly successfully. It is clear that we need time for this, but investing in this gives better results in the end... "*
	Perception of a positive impact in the patient	21	**P131:** *"It is good to get the patients to participate in their process, since by assuming their part of the responsibility it will help them to better understand their illness and face it better."*<br>**P77:** *“The patient becomes the true protagonist of their own treatment, taking responsibility for sticking to it; the patient begins to have a sense of control and co-responsibility. "*
	Perception of improvement in the process	19	**P16:** *"I totally agree. Informed patients, capable of making decisions and managing their disease help to improve the health system and their own health. We would reduce waiting lists; we would decongest accident and emergency departments.*
			**P133:** *“When one is aware of the objectives, and knows and controls the disease, relying on the doctor for decision-making, this does not only provide benefits at the health level, but also means the patient is aware and takes part at the level of health spending, and control of resources.*
			P115: "At this point, at some point I still get caught up with certain paternalistic attitudes, ... inertia, hyper-concentrated consultations ... there are many conditioning factors ... However, consultations flow better, the moment the patient feels they have the ability to decide regarding their processes, it increases confidence and provides me with more information, treatments are more individualized, better adherence to treatments, more modifications in lifestyle ... and of course greater satisfaction for both me as a professional as well as for patients. "
