# Supplementary material for: Healthcare Professionals’ Perceptions about the Implementation of Shared Decision-Making in Primary Care: A Qualitative Study from a Virtual Community of Practice
Source: Int J Integr Care. 2024 Apr 16;24(2):8. doi: 10.5334/ijic.6554 (PMC11025573; doi:10.5334/ijic.6554)
Supplement: Appendix 4. — Screenshot of the Screenshot of the e-MPODERA project’s Virtual Community of Practice. [file ijic-24-2-6554-s4.pdf]

Appendix 4. Screenshot of the Screenshot of the e-MPODERA project's Virtual Community of Practice

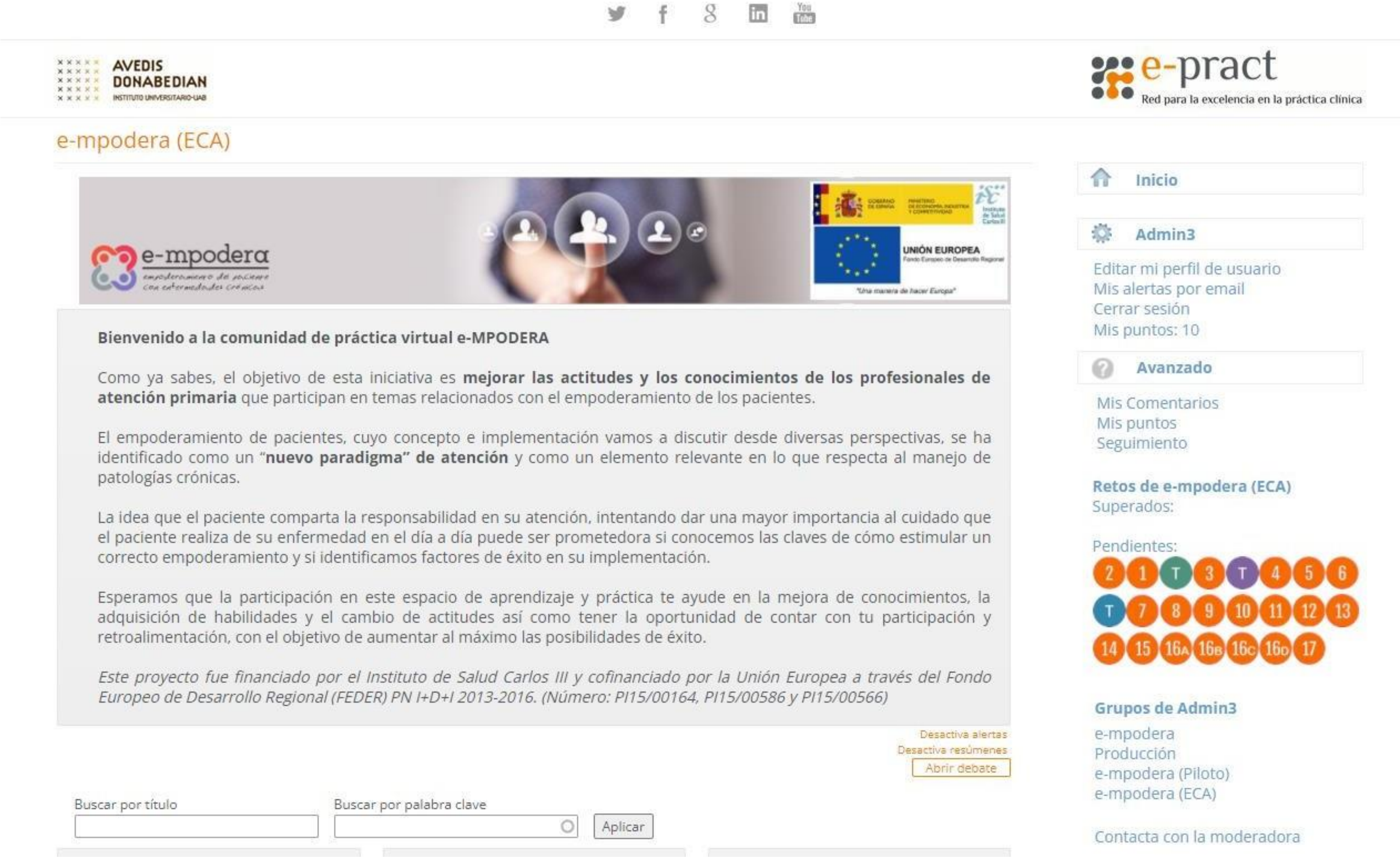

¡e-mpodera ha finalizado!

¡Gracias a tod@s por participar de e-mpodera! La plataforma permanecerá abierta aunque no se agregarán contenidos nuevos en los próximos días....

5 comentarios

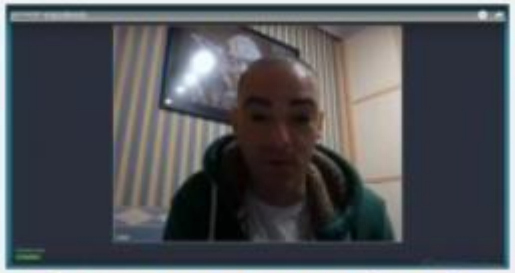

Webinario con Oscar López de Briñas (e-mpoderado!)

EMPODERAMIENTO; PACIENTE EXPERTO

¡Gracias Oscar por compartir tu experiencia! Aquí compartimos el webinario para todos los que no han podido participar.

11 comentarios

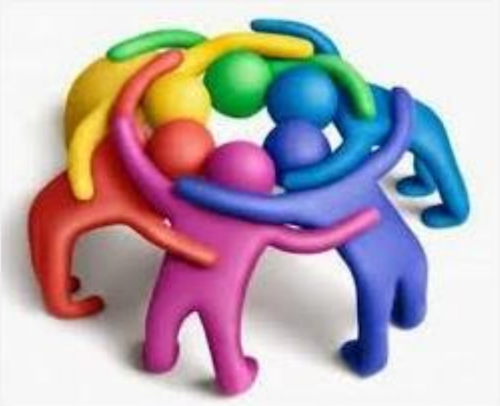

Reto 15: Colaborativo! Talleres de empoderamiento grupal

EMPODERAMIENTO, EDUCACION GRUPAL, TALLERES, ENFERMEDADES CRÓNICAS

15

Durante los meses que llevamos de e-mpodera hemos adquirido muchos recursos que hemos puesto en práctica de manera individual a través de diferentes retos. ...

56 comentarios

102 respuestas

Más días para finalizar tus retos!!

e-mpodera finaliza, pero te damos más días para que puedas finalizar los retos pendientes y participes de los últimos retos colaborativos (16A, 16B, 16C y 16D) para que...

11 comentarios

Reto 16A: Obesidad, diabetes mellitus

RETO, DIABETES, EMPODERAMIENTO, EDUCACION GRUPAL, ENFERMEDADES CRÓNICAS

16A

En el siguiente documento colaborativo tenéis que realizar una propuesta de taller grupal para facilitar que las personas que lo realicen estén más activadas, informadas...

52 comentarios

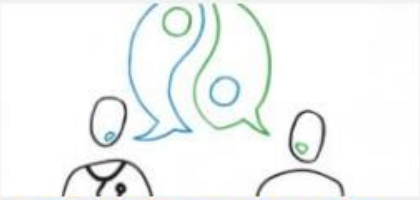

Reto 14: ¡Practicando la Toma de Decisiones Compartidas!

HERRAMIENTAS PARA LA TOMA DE DECISIÓN COMPARTIDA, DECISIONES COMPARTIDAS, RETO

14

En este nuevo reto vamos a implementar lo que hemos venido aprendiendo sobre Toma de Decisiones Compartidas. 1) Se trata de que escojáis un...

76 comentarios

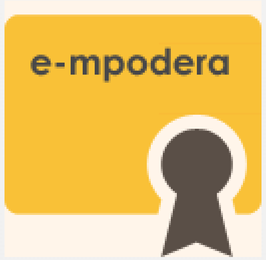

RETO 17. EMPODERAMIENTO GRUPAL: votamos el proyecto más completo e innovador!

EMPODERAMIENTO, EDUCACION GRUPAL, TALLERES, ENFERMEDADES CRÓNICAS

17

¡Ya habéis finalizado los talleres! ¡Enhorabuena por el buen trabajo realizado! En este último reto elegiremos el taller más completo e innovador....

59 respuestas

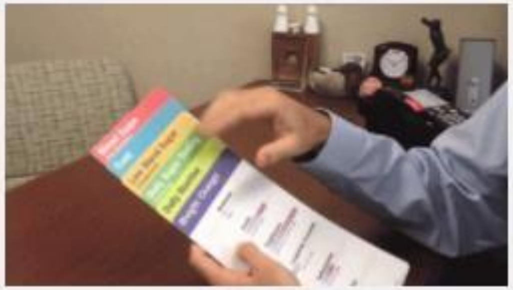

Herramienta para la toma de decisiones compartidas

DECISIONES COMPARTIDAS, DIABETES, HERRAMIENTAS PARA LA TOMA DE DECISIÓN COMPARTIDA

¿Qué te parece este vídeo? ¿Aplicarías una herramienta así con tus pacientes? Coméntalo en el Debate!

15 comentarios

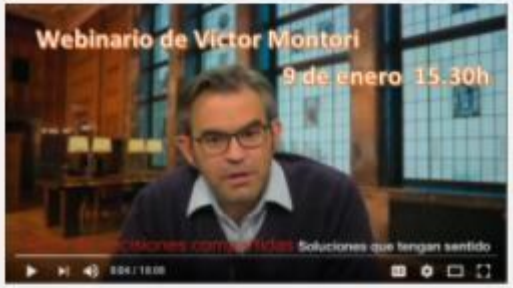

Video de Victor Montori para el Webinario de Toma de Decisiones Compartidas

DECISIONES COMPARTIDAS

En este vídeo Victor Montori hace un recorrido por diferentes modelos que se han propuesto para entender la Toma de Decisiones Compartidas.   Nos aporta...

10 comentarios

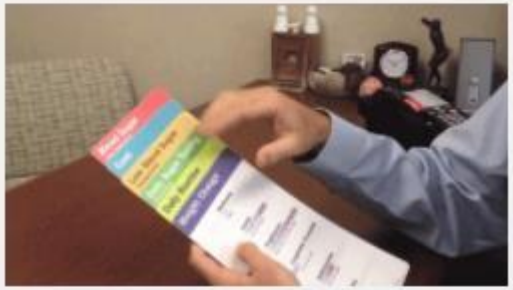

Herramienta para la toma de decisiones compartidas

DECISIONES COMPARTIDAS, DIABETES, HERRAMIENTAS PARA LA TOMA DE DECISIÓN COMPARTIDA

¿Qué te parece este vídeo? ¿Aplicarías una herramienta así con tus pacientes? Coméntalo en el Debate!

15 comentarios

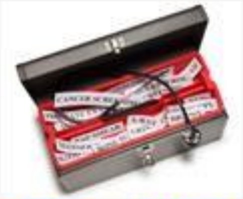

Reto 12. Entendiendo mejor las decisiones compartidas… y a buscar herramientas!

DECISIONES COMPARTIDAS, HERRAMIENTAS

12

ENTENDIENDO MEJOR LAS DECISIONES COMPARTIDAS… Continuando con el Reto 11: comenzando a hablar de decisiones compartidas, cada una de las definiciones que…

105 comentarios

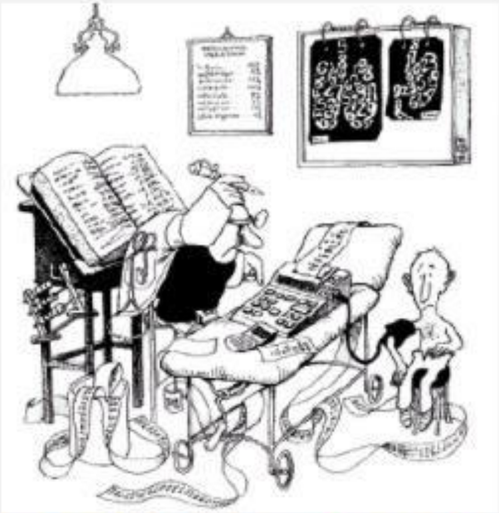

Reto 7: Actitudes profesionales que DESempoderan

RETO, ACTITUDES, DESEMPODERAMIENTO, EMPODERAMIENTO

7

En este nuevo reto, reflexionaremos sobre actitudes de los profesionales que DESEMPODERAN a los pacientes. Para esto, vamos a pensar o buscar ejemplos…

190 comentarios

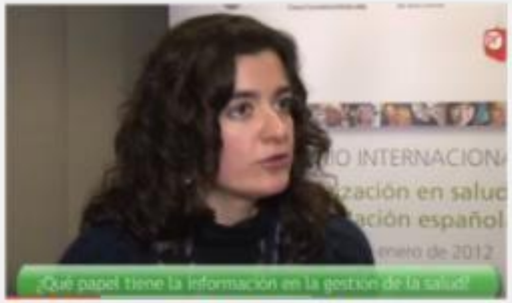

Más sobre alfabetización sanitaria

ALFABETIZACIÓN SANITARIA, INFORMACIÓN, INTERNET

Continuamos avanzando con el tema de alfabetización sanitaria y conociendo las diferentes fuentes de información sanitaria que utilizan las personas que…

7 comentarios

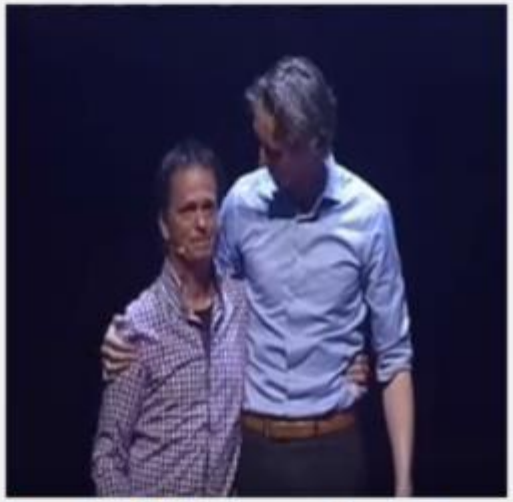

Reto 2: ¿Trabajamos juntos?

EMPODERAMIENTO, RETO

2

Después de ver este vídeo reflexiona y contesta a la siguiente pregunta: ¿Creeis que a veces "Desempoderamos a nuestros pacientes" ? ¿Tenéis ejemplos de frases...

173 comentarios

Reto 16B: Hipertensión, cardiopatía isquémica, insuficiencia cardíaca

RETO, ENFERMEDADES CRÓNICAS, INSUFICIENCIA CARDÍACA, EDUCACION GRUPAL, EMPODERAMIENTO

16B

En el siguiente documento colaborativo tenéis que realizar una propuesta de taller grupal para facilitar que las personas que lo realicen estén más activadas,...

44 comentarios

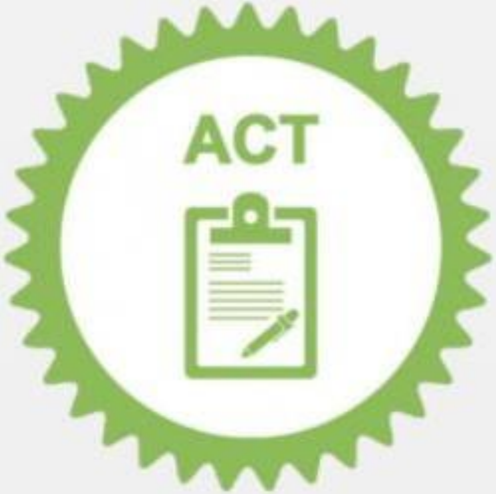

Reto 9: Implementando las acciones planificadas

RETO, ACCIÓN DE MEJORA, OBJETIVOS, EMPODERAMIENTO

9

Aunque aún tenemos un reto activo (que se sugiere acabar el 20 de Julio) hemos abierto el reto de acción para aquellos que ya habéis acabado la planificación. Para...

91 comentarios

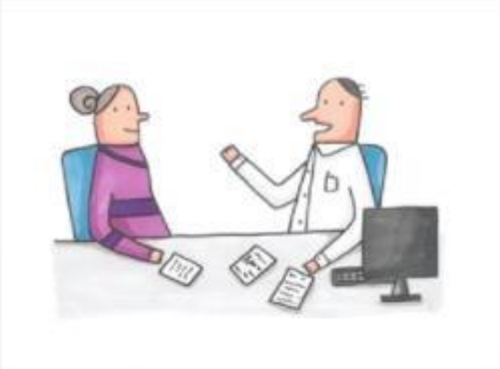

Reto 11: Comenzando a hablar de decisiones compartidas

DECISIONES COMPARTIDAS

11

En las últimas semanas hemos ido trabajando juntos diversos conceptos que se relacionan con el empoderamiento, por ejemplo la activación. Hemos pensado sobre cómo...

81 comentarios

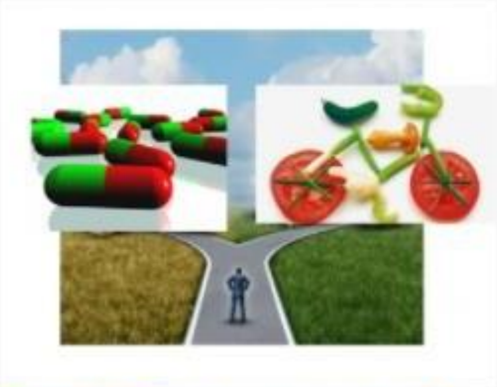

Reto 13: ¿Tratar o no tratar? Tomando Decisiones Compartidas con José

ACTIVACIÓN, COMUNICACIÓN, ALFABETIZACIÓN SANITARIA, DECISIONES COMPARTIDAS, HERRAMIENTAS PARA LA TOMA DE DECISIÓN COMPARTIDA

13

Por tanto, este nuevo reto requiere DESARROLLAR LA ENTREVISTA (puedes escribir el diálogo que imagines) y VOTAR aquellas entrevistas de vuestros compañer@s que os...

91 comentarios

Reto 16D: Paciente anciano, prevención de caídas, AVC, demencias, cuidado del cuidador

RETO, ENFERMEDADES CRÓNICAS, EDUCACION GRUPAL, EMPODERAMIENTO

16D

En el siguiente documento colaborativo tenéis que realizar una propuesta de taller grupal para facilitar que las personas que lo realicen estén más activadas,...

68 comentarios

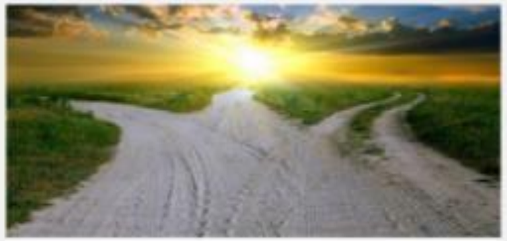

Reto 10: Caso ¿Te acuerdas de Sara?

ACTIVACIÓN, COMUNICACIÓN, ENTREVISTA MOTIVACIONAL, DIABETES, ALFABETIZACIÓN SANITARIA

10

Por tanto, este nuevo reto requiere DESARROLLAR LA ENTREVISTA con todas las personas implicadas teniendo en cuenta los 5 puntos anteriores (puedes escribir el...

113 comentarios
